# Can Selenium Reduce the Susceptibility and Severity of SARS-CoV-2?—A Comprehensive Review

**DOI:** 10.3390/ijms23094809

**Published:** 2022-04-27

**Authors:** Muhammed Majeed, Kalyanam Nagabhushanam, Priji Prakasan, Lakshmi Mundkur

**Affiliations:** 1 Sami-Sabinsa Group Limited, 19/1&19/2, I Main, II Phase, Peenya Industrial Area, Bangalore 560-058, Karnataka, India; mail1@sami-sabinsagroup.com (M.M.); priji@sami-sabinsagroup.com (P.P.); 2 Sabinsa Corporation, 20 Lake Drive, East Windsor, NJ 08520, USA; kalyanam@sabinsa.com

**Keywords:** selenium, micronutrient, SARS-CoV-2, immunity, inflammation, oxidative stress

## Abstract

The SARS-CoV-2 infection is a highly contagious viral infection, which has claimed millions of lives in the last two years. The infection can cause acute respiratory distress, myocarditis, and systemic inflammatory response in severe cases. The interaction of the viral spike protein with the angiotensin-converting enzyme in various tissues causes damage to vital organs and tissues, leading to complications in the post-infection period. Vaccines and antiviral drugs have improved patient response to the infection, but the long-term effect on vital organs is still unknown. Investigations are now focused on supportive nutrient therapies, which can mitigate the susceptibility as well as the long-term complications of COVID-19. Selenium is one such micronutrient that plays a vital role in preventing oxidative stress induced by the virus. Further, selenium is important for effective immune response, controlling systemic inflammation, and maintain overall health of humans. We examine the role of selenium in various aspects of SARS-CoV-2 infection and address the importance of selenium supplementation in reducing the susceptibility and severity of infection in this review.

## 1. Introduction

In 2019, pneumonia of an unknown origin was reported in Wuhan, Hubei province of China, which was a tocsin to warn of a pandemic. The pneumonia cases were pathologically linked to a coronavirus, a novel beta-coronavirus belonging to the subgenus Sarbecovirus and was designated as ‘severe acute respiratory syndrome coronavirus 2’ (SARS-CoV-2) [1]. Coronavirus disease 2019 (COVID-19) has since spread worldwide, pressing the World Health Organization to declare it as a pandemic on 12 March 2020 [2]. The symptoms of COVID-19 range from mild to severe, while a large portion of infected people remain asymptomatic or mildly symptomatic carriers. The risk and severity of infection is higher in individuals with comorbidities such as diabetes, obesity, hypertension, cardiovascular and respiratory diseases [3]. Generally, COVID-19 is characterized by fever, tiredness, cough, and other respiratory and gastrointestinal issues [4]. The rapid transmissibility and wider community spread of COVID-19 may be attributed to the uncontrolled replication of SARS-CoV-2, the higher binding efficiency of its spike protein to human cell receptors, and its efficiency in overruling the host immune defense [5,6]. Although the major target of SARS-CoV-2 is the lungs, its systemic effects may lead to devastating effects on other vital organs such as the kidney, heart, liver, eye, and brain [7]. To date, SARS-CoV-2 has affected over 200 countries and territories, causing several millions of deaths worldwide [8].

**Figure 1:**
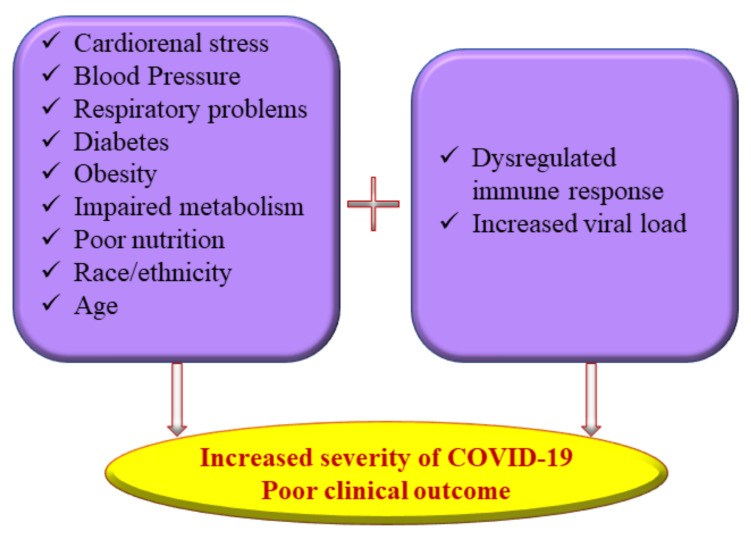
Multiple risk factors of severe coronavirus disease 2019 (COVID-19) illness.

Considerable time and effort were spent tracking, isolating, and treating COVID-19 patients in the early stages of the pandemic, which piled up an irrevocable socio-economic burden on several countries worldwide [9]. Vaccines, antiviral drugs, and repurposed drugs continue to be used with different levels of efficacy to combat the new variants of SARS-CoV-2 emerging every few months [10,11,12,13]. The current research approach has seen a surge in interest in identifying the epidemiological factors underlying the susceptibility to COVID-19 and to exploring novel preventive and curative options. As the pandemic unfolds, it is evident that several physiological factors, including malnutrition, exacerbate the susceptibility and severity of coronavirus infections similar to any other viral disease (Figure 1).

## 2. Nutrition and COVID-19

Malnutrition is a state of nutrient inadequacy attributed to insufficient energy and health issues and is usually associated with multiple micronutrient deficiencies. Nutritional supplementation may reduce the susceptibility to infection, prevent severe disease, and help patients recover faster with milder post-recovery morbidities [14]. Micronutrients such as vitamins and trace elements such as iron, zinc, copper, selenium, and omega-3-polyunsaturated fatty acids are essential for the healthy functioning of the immune system [15]. 

Micronutrient deficiencies or suboptimal status may weaken immune responses and decrease resistance to infections. A study conducted in COVID-19 patients of China showed that poor nutritional status is a predisposing factor leading to higher disease severity. The prognostic nutritional index (PNI) score was found inversely related to the severity of COVID-19, and it significantly decreased in patients with common to severe forms of COVID-19 regardless of sex, age range, and body mass index (BMI) [16].

Lack of proper pharmacological treatment for COVID-19 has encouraged alternative therapies to reduce the symptoms or severity of COVID-19 [17]. Several observational studies have highlighted the association of nutrients with the altered immune response and increased severity and mortality of COVID-19 [15,18,19]. Evidence-based studies suggest that nutritional status, including the level of micronutrients, plays an important role in the progression of COVID-19, where the selenium status of the body is ineludible [18].

Selenium is an indispensable trace element required for the body’s normal functioning that imparts its physiological role via selenium-containing proteins called selenoproteins. Selenium is involved in innate and acquired immunity, including T and B lymphocyte function, and is necessary for antibody production and thus influences susceptibility to infections [19]. The primary source of selenium is from the diet, which in turn depends on the soil selenium content and its bioavailability to crops [20]. Numerous reports explain the beneficial effects of selenium in several human ailments such as cardiovascular diseases, obesity, diabetes, cancer, and microbial infections [21].

## 3. Mechanism of SAR-CoV-2 Infection

Coronaviruses belong to crown-shaped animal viruses that mainly affect the respiratory system. The symptoms vary from the common cold to severe respiratory diseases. The Middle East Respiratory Syndrome virus (MERS-CoV) and severe acute respiratory syndrome virus (SARS-CoV) were the most recent infections caused by coronaviruses before COVID-19. Several canine and feline coronaviruses are present in animals, but they are harmless to humans, as they are not transmitted from animals to human beings. However, in rare cases, coronaviruses start to infect humans similar to what happened in the case of COVID-19, caused by a novel coronavirus, SARS-CoV-2 [22]. 

**Figure 2:**
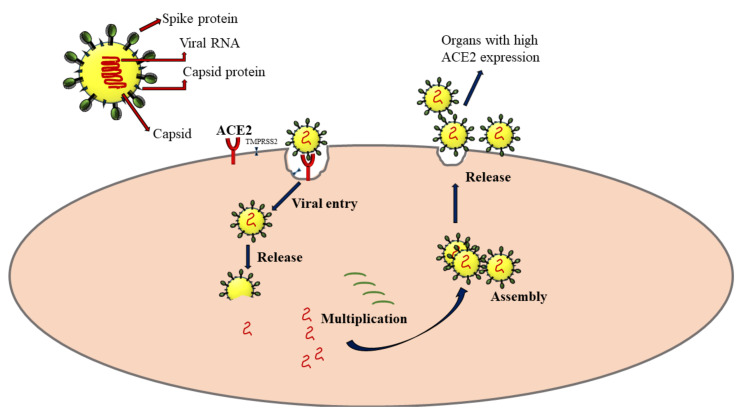
Mechanism of SARS-CoV-2 infection via the interaction with angiotensin-converting enzyme 2 (ACE2) receptors. TMPRSS2: Transmembrane protease, serine 2. The arrows represent the sequence of events following SARS-CoV-2 infection.

In humans, SAR-CoV-2 primarily affects the respiratory system, especially the alveolar epithelial cells in lung sacs. The crown-like spike proteins on the surface of viruses help them complex with the receptors such as angiotensin-converting enzyme 2 (ACE2) [23]. After its entry into the host cell, it utilizes the host cellular machinery for synthesizing its genome materials and proteins. The virus progeny is released, which can infect new cells to propagate the infection (Figure 2). The extent of cellular damage is a balance between host immunity and the ability of the virus to escape this defense. The severity of COVID-19 varies widely from asymptomatic and mildly symptomatic to deadly, critically depending on several pathophysiological conditions of the host. The ACE2 is widely expressed in lung, heart, kidney, neuronal, gastrointestinal, and liver cells [24]. Severe SARS-CoV-2 infection has the potential to intrude on these tissues and damage these organs, leading to multiple organ damage [25]. It is now proven that COVID-19 infection dysregulates cytokine homeostasis, leading to cytokine storm, characterized by severe systemic elevation of several pro-inflammatory cytokines. A cytokine storm can be edematous, causing acute respiratory distress syndrome (ARDS), pneumonia, and multiple organ failure in some patients [26]. 

## 4. Selenoproteins in SAR-CoV-2 Infection

Proteins with the 21st amino acid, L-selenocysteine (Sec), are known as selenoproteins. Selenocysteine is encoded by the genetic codon UGA, which is otherwise a stop codon [27]. During translation, Sec is co-translationally incorporated into a growing polypeptide chain with the assistance of unique elements such as Sec insertion sequence (SECIS), Sec-specific elongation factor (eEFsec), its own transfer RNA (tRNA[Ser]^Sec^), the specific binding proteins for Sec insertion (SBP2), ribosomal protein L30, and nucleolin, to ensure the correct insertion of Sec into selenoproteins [28,29]. Around 25 selenoprotein genes have been characterized in humans, and some of these proteins play a pivotal role in maintaining redox homeostasis, modulating immune response and regulating inflammatory cascade [30]. 

### 4.1. Glutathione Peroxidases (GPxs)

Glutathione peroxidases are involved in oxidative homeostasis and play a crucial role in viral pathogenesis. GPx1-4 and 6 are the five GPx isoforms that contain Sec in humans. GPxs catalyze the reduction of hydrogen peroxides using glutathione (GSH) as a cofactor to prevent oxidative stress. [31]. They also regulate the redox signaling pathways involved in cell proliferation, differentiation, apoptosis, cytokine expression, ultimately affecting the overall health. Gpx1 and GPx4 are the most abundant proteins, while GPx3 is a glycosylated protein secreted in the plasma, whose activity correlates with the selenium status of the organism. GPx2 is present in epithelial tissues, and GPx6 is mainly associated with embryonic tissue and olfactory cells [30].

GPx1 is a predominantly cytosolic protein with antiviral properties. Gene knockout studies in mice have shown that the absence of GPx1 overwhelms oxidative stress, resulting in enhanced pathogenicity and virulence of viruses. Lack of Gpx1 led to viral mutation and induced myocarditis in knockout mice compared to the wild type [32]. A selenium-deficient diet increased the mutagenicity and virulence of benign coxsackievirus B3, suggesting the importance of selenium in GPx1 activity. Both selenium deficiency and GPx1 knockout heightened inflammatory responses and more severe lung pathology in response to influenza A infection, further indicating that GPX1 participates in molecular mechanisms protecting against viral infections of the respiratory tract [33]. Wang et al. (2020) reported that GPx1 activity is reduced during COVID-19, affecting the expression of other selenoproteins. Reduced GPx1 level concurrently increases intracellular ROS and membrane lipid peroxidation in experimental studies performed with Se-deficient Vero E6 cells infected with SARS-CoV-2. These results emphasized the significance of Se for its potential use against COVID-19 [34]. The interaction between GPx1 and M^pro^, the main protease necessary for viral replication, is currently gaining attention as a potential therapeutic agent for COVID-19. Ebselen, a selenium-containing GPx1 mimetic, has emerged as the strongest inhibitor of M^pro^ and a target of great interest for therapeutic interventions for COVID-19 [35]. 

Obesity is an important risk factor exacerbating the severity of COVID-19, which is often associated with cardiovascular disorders and thrombotic complications in critically ill SARS-CoV-2 patients [36]. Obesity was found to decrease the activity of selenoproteins such as GPx3 in adipose tissues, of which the supplementation of selenium could improve. Thus, selenium supplementation was an effective supportive therapy for preventing cardiovascular impairments in obese COVID-19 patients admitted to the intensive care unit [37]. 

SARS-CoV-2 infection is known to increase the production of mitochondrial ROS, helping viral replication [38]. Of the different GPx isoforms, only GPx4 is located in the mitochondria, suggesting that targeting GPX4 increase may be beneficial. Currently, different mechanisms are being proposed for the role of GPx4 in COVID-19. Ferroptosis is a mechanism of programmed cell death caused by lipid-reactive oxygen species accumulation due to ferric ion overload in cells resulting in lipid peroxidation [39]. Inactivation of GPx4 and glutathione depletion are considered as the major leading causes of ferroptosis, as GPx4 protects the cell membrane against lipid peroxidation and ferroptosis by eliminating lipid ROS [40,41]. GPx4 specifically protects the cells of the immune system against ferroptosis cell death. It is also proposed that leukopenia in COVID-19 patients may be associated with SARS-CoV-2-induced ferroptosis [42]. Further, GPx4 is also essential for activating the stimulator of interferon genes, which is pivotal in initiating innate immune responses against viral infection [42]. The findings also emphasize the importance of selenium compounds in the prophylaxis and treatment of COVID-19. 

Infections of the olfactory epithelium result in a loss of smell in COVID-19 patients, one of the characteristic symptoms of SARS-CoV-2 infection. GPx6 is localized to the olfactory epithelium and is involved in scavenging hydrogen peroxide and hydroperoxides in humans and mice [29]. It is proposed that Se supplementation could reduce olfactory mucosal inflammation, preventing viral infection/replication, including SARS-CoV-2.

### 4.2. Thioredoxin Reductases (TXNRDs)

Thioredoxin reductases (TXNRDs) are a family of selenoproteins that reduce thioredoxins and other endogenous and exogenous substrates, thereby controlling the expression of genes involved in cell growth, proliferation, and inflammation. Thioredoxins are necessary for DNA synthesis, as it converts ribonucleotides to deoxyribonucleotides. They are synthesized in response to oxidative stress and are upregulated by the nuclear factor-erythroid 2 related factor 2(NRF2), a cytoprotective transcription factor. Thioredoxins reduce oxidative stress and prevent viral propagation [42]. Experimental evidence indicates that selenium-deficient Vero E6 cells infected with SARS-CoV-2 significantly downregulated TXNRD3 [34].

### 4.3. Selenoprotein P (SELENOP)

SELENOP is one of the major circulatory antioxidant selenoproteins in plasma that helps to transport selenium to specific tissues. Moghaddam et al. (2020) observed lowered serum selenium and SELENOP concentrations in COVID-19 patients in Germany. The patients who died showed a significantly greater deficiency of selenoproteins, including SELENOP, than those who survived [43]. Severe hypoxia and an increase in IL6, the hallmark of SARS-CoV-2 infection, are the other factors that suppress hepatic SELENOP expression, reducing circulating SELENOP levels and selenium delivery to tissues [44,45]. It was suggested that reducing the deficit of selenium and SELENOP in SARS-CoV-2-infected patients through supplementation could enable enhanced redox control and response to the immune system in COVID-19 management. 

### 4.4. Other Selenoproteins

Recently, infection of selenium-deficient Vero E6 cells with SARS-CoV-2 revealed a downregulation of SELENOF, SELENOK, SELENOM, and SELENOS localized in the ER, indicating that infection could adversely affect ER [34]. Compromised SELENOF and SELENOM proteins increase protein misfolding, while ER-associated degradation of misfolded proteins is attenuated by impaired SELENOK and SELENOS expression [29]. Wang et al. found a profound reduction of SELENOF, which is targeted for proteolysis by the SARS-CoV-2 main protease M^pro^ [46]. A large cohort study found a significantly higher risk of COVID-19-related death in African American ethnicity, which also harbors a polymorphic site at nucleotide position 1125 (A/G) located in the SECIS element of SELENOF, affecting the efficiency of selenocysteine incorporation into SELENOF in a Se-dependent fashion [47,48]. 

## 5. Role of Selenium on the Management of Viral Infection

**Figure 3:**
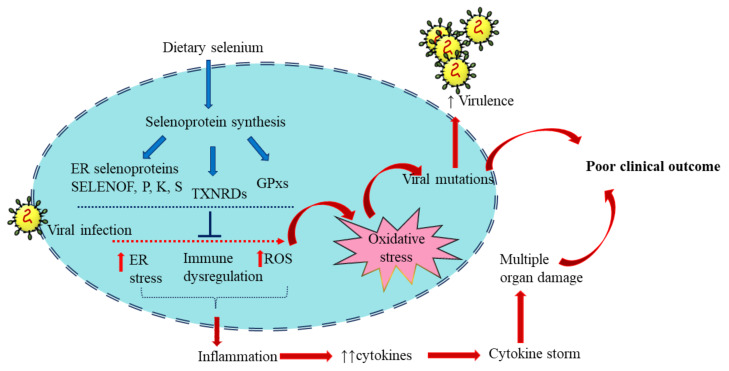
Selenoproteins in viral infection: Dietary selenium is incorporated in selenoproteins that play regulatory roles in immune function and redox homeostasis. These include the Selenoproteins glutathione peroxidases (GPX), 15 kDa selenoprotein F (SELENOF), selenoproteins K and S and thioredoxin reductases (TXNRD). Viral infection alters the expression of selenoproteins and induces oxidative stress, increasing virus virulence. The resultant inflammation and excess cytokine production eventually lead to poor clinical outcomes. The blue arrows represent the sequence of events of selenoprotein synthesis from dietary selenium. The red arrows represent the consequence of viral infection. Adequate selenium levels can reduce the impact of viral infection and help in an optimum antiviral response.

Selenium plays a vital role in managing viral infections through antioxidant, inflammatory, and immunomodulatory mechanisms to reduce the adversity of clinical manifestations (Figure 3).

### 5.1. Selenium in Oxidative Stress

As discussed in an earlier section, selenoproteins are involved in several cellular functions, including antioxidant defense, cell signaling, and redox homeostasis, which are important in viral pathogenesis [30]. Every respiring cell produces reactive oxygen species during mitochondrial oxidative phosphorylation. Generally, in a healthy system, a balance is maintained between the generation and scavenging of ROS where selenoproteins such as glutathione peroxidases and thioredoxin reductases are essential [49]. Moreover, ER selenoproteins and methionine sulfoxide reductase play crucial roles in protein folding and stress response [29]. Viral infections, especially respiratory viral diseases, are characterized by a broad spectrum of clinical symptoms, with oxidative stress being one of their hallmarks. The pathophysiology of viral infections reveals the alteration in intracellular redox status in hosts with impaired immune response. Selenoproteins reduce the oxidative stress generated by viral infections such as human immunodeficiency virus (HIV), hepatitis B/C viruses, Epstein–Barr virus (EBV), herpes simplex virus type 1 (HSV-1), influenza viruses, vesicular stomatitis virus (VSV), respiratory syncytial virus (RSV), and human T cell leukemia virus type 1 (HTLV-1) [30,50]. Selenium deficiency induces oxidative stress in the host that can alter the viral genome, making it more virulent and pathogenic [51]. 

Some low molecular weight selenium compounds such as dimethyl selenides, methyl selenol, and the intermediates of selenium metabolism, including dimethyl diselenide and selenodiglutathione, were shown to have strong redox activity [52]. These compounds were reported to modify the Cysteine 145 residue of SARS-CoV-2 M protein and prevent viral replication [53].

### 5.2. Selenium in Inflammation

Inflammation is an important defensive mechanism of the body against an external stimulus such as viral infections. Several cases of COVID-19 have reported higher elevated levels of some plasma cytokines. Most of the critical illnesses of COVID-19 are associated with a hyperinflammatory process called hypercytokinemia or cytokine storm [26]. COVID-19 patients admitted to ICU showed unregulated cytokine production characterized by higher levels of granulocyte–macrophage colony-stimulating factor (GM-CSF), interferon gamma-induced protein 10 (IP10), monocyte chemoattractant protein-1 (MCP-1), macrophage inflammatory protein 1 alpha (MIP1A), and tumor necrosis factor-alpha (TNFα) compared to non-ICU patients [46]. C-reactive protein, ferritin, IL-6, and other inflammatory indices were significantly high in critically ill patients in China [54]. The hyperinflammatory responses and cytokine storm force the immune cells to destroy healthy cells, leading to epithelial damage, hyaline membrane formation, thrombus formation, and vascular leakage, which result in lung damage and ARDS [55]. Approximately 33% of the hospitalized COVID-19 patients were reported to have ARDS [56,57]. A retrospective study in patients admitted to the intensive care unit (ICU) with confirmed COVID-19 showed highly elevated levels of IL-6 and delayed cytotoxic immune responses, which are characteristics of severe COVID-19-induced ARDS [58]. Many families of viruses, including HIV, H1NI, hepatitis virus, are known to activate NF-κB, promoting viral replication and preventing virus-induced apoptosis. Se/selenoproteins can decrease activation of NF-κB, reducing viral replication [53]. 

Intravenous selenium supplementation in the form of sodium selenite to critically ill patients with ARDS showed replenished selenium status, restored the antioxidant capacity of the lungs, and moderated inflammatory responses suppressing IL-1β and IL-6 levels. Selenium supplementation improved the total respiratory mechanics, reducing ARDS [59]. Selenium supplementation upregulated the anti-inflammatory responses in lipopolysaccharide-stimulated murine macrophages [60]. Preclinical and clinical studies have proven the immune-modulatory effects of dietary selenium supplementation, such as elevating the transcription of anti-inflammatory selenoenzymes (GPx) and downregulating the expression of pro-inflammatory inducible enzymes, pro-inflammatory cytokines (IL-1, IL-2, IL-6, TNF-α), and chemokines [61]. An expert panel in Iran designed an internal protocol to manage COVID-19-induced ARDS according to WHO recommendations and NIH guidelines, which included the supplementation of vitamins and selenium at a dosage of 200 µg/day along with standard therapy. Selenium supplementation was found effective in regulating oxidative stress pathways and ARDS [62]. 

### 5.3. Selenium and Immunity

Selenoproteins are important for immunity and inflammation, as they are necessary for the production and maturation of antibodies [63,64]. Animal studies have shown that selenium deficiency adversely affects innate and acquired immunity components, including T-cell/macrophage-dependent antibody production and susceptibility to infections [65]. Selenoprotein-deficient T cells and macrophages showed elevated ROS and oxidative stress in mice [66]. The level of mature and functional T-cells in lymphoid tissues was reduced, resulting in the impairment of T-cell-dependent antibody responses. Conversely, selenium deficiency did not influence the normal immune responses of macrophages but reduced the invasiveness that impaired the migration of macrophages through the extracellular matrix and basement membrane [66]. Selenium supplementation modulated T-lymphocyte-mediated immune responses in humans as well. Selenium supplementation augmented the ability of peripheral blood lymphocytes to respond to stimulation by enhancing the expression of high-affinity IL-2 receptors on their surface [67]. 

Selenium is required for the function of CD8+ T cytotoxic cells and natural killer (NK) cells. Selenium enhanced the lytic activity of activated natural killer cells. It also boosted the proliferation, expansion, and lytic activity of lymphokine-activated killer cells by increasing the expression of intermediate affinity IL-2 receptors on these cells [68]. Moreover, selenium supplementation restored the inability of spleen lymphocytes to respond to stimulation by enhancing nuclear DNA synthesis and cell proliferation in mice. Selenium supplementation was shown to influence the proliferation and differentiation of CD4+ T cells to Th1 compared with Th2 effector cells, enhancing the antiviral responses [69]. Similarly, a high-dose supplementation of selenium, 200 µg/day for nine months, was found effective in reducing the progression of HIV viral load and elevating the serum selenium level in HIV-1-seropositive men and women. It also improved the CD4+ cell count in the study subjects. Although the exact mechanism that selenium exerts on viral replication is not known, several studies have proposed that the antioxidant property may be one of the prominent contributing factors to antiviral effects [70]. 

### 5.4. Selenium in Thromboembolism

SARS-CoV-2 infection not only affects the respiratory system but also impart its adverse events in other vital organs in susceptible patients. The inflammatory chemicals and cytokine storm induce the liver to produce defensive proteins that can cause blood clotting, resulting in thromboembolism. It clogs the blood vessels and blocks the supply of nutrients and oxygen to organs such as the heart, brain, and kidney, leading to multi-organ failure and death [71]. The viral infections inducing thromboembolism have the risk of developing poor prognostic events such as stroke, plaque instability, vasculitis, myocardial infarction, stroke, necrotizing encephalitis, and myalgia in critically ill patients [55]. Ebselen, an organoselenium compound, showed significant improvement in patients diagnosed with acute ischemic stroke at a dose of 150 mg twice per day for two weeks [72]. In another study, Ebselen was shown to be effective against focal ischemic injury induced by photothrombosis (a form of ischemic damage to the brain that results in a stroke) in rats by decreasing IL-6 [73]. Supplementation of 200 µg/day of organic selenium yeast tablets along with coenzyme Q10 prevented an increase in D-dimer, a biomarker of venous thromboembolism, and reduced the risk of cardiovascular mortality in older adults in comparison with the placebo group [74]. Similar beneficial effects of selenium on thromboembolism were also observed in patients with myocardial infarction [75]. 

## 6. Clinical Studies on Selenium and COVID-19

### 6.1. Selenium Deficiency in COVID-19 Patients

Selenium deficiency is reported to affect 500 million to 1 billion people worldwide, mainly due to inadequate dietary intake of selenium. The baseline immune capabilities are affected by the intake of selenium. Several observational studies have revealed the association between low selenium status and increased susceptibility to viral infections. Low selenium level reduces immunity and enhances the multiplication of viruses. Selenium intake varies in different regions of the world. China is one of the most selenium-deficient countries, with populations bearing the lowest and highest selenium status. An observational study was conducted in two cities of China with two extremes of selenium status to analyze the association of selenium status and COVID-19. The cure rate was higher in Enshi city, renowned for its high selenium intake and status [76]. The selenium levels in hair were 3.13 ± 1.91 mg/kg for females and 2.21 ± 1.14 mg/kg for males in Enshi city, compared to 0.55 mg/kg in Hubei cities. Similar effects were observed in Heilongjiang province, a notoriously low-selenium region that experienced a much higher death rate and low cure rate compared to the other provinces. Hence, the study suggested a strong correlation between viral replication, pathogenicity, or mortality with selenium deficiency, as reported here for SARS-CoV-2 [77]. A study assessed the nutritional status of 50 hospitalized patients with COVID-19 in South Korea, revealing that almost 42% were selenium deficient. Thus, the study highlighted that nutritional deficiencies of selenium and vitamin D might possibly favor the onset of COVID-19 and increase the severity of the disease [78]. An ecological study was conducted in China to assess the association between the COVID-19 case fatality rate (CFR) and the selenium content, both from crops and topsoil. Of the 14,045 COVID-19 cases reported from 147 cities, based on selenium content in crops, the CFRs gradually increased from 1.17% in non-selenium-deficient (selenium adequate) areas to 1.28% in moderate-selenium-deficient areas, and severe selenium-deficient areas were affected severely with a CFR of 3.16%. Similarly, based on selenium content in topsoil, the CFRs gradually increased from 0.76% in non-selenium-deficient areas to 1.70% in moderate-selenium-deficient areas, and further to 1.85% in severe selenium-deficient areas. Thus, the results suggest that regional selenium deficiency can be related to an increased CFR in COVID-19 [79]. A cross-sectional comparative study was conducted in 50 patients with COVID-19 symptoms admitted to an isolation center in Northwestern Nigeria. The trace mineral antioxidant levels such as selenium (Se), zinc (Zn), magnesium (Mg), copper (Cu), and chromium (Cr) were measured with 8-isoprostaglandin F2 alpha, malondialdehyde glutathione activity in erythrocytes and activities of glutathione, glutathione peroxidase, superoxide dismutase and catalase. The plasma selenium, GPx and vitamins (A, C, and E), Zn, and Mg levels were significantly lower in COVID-19 patients than in healthy individuals. Oxidative stress markers, 8-isoprostaglandin F2 alpha, were significantly higher in COVID-19 patients than controls. These observed lower values of antioxidant vitamins and enzymes, including selenoproteins in COVID 19 subjects, may be due to overproduction of ROS, and they are being depleted in the process of scavenging these radicals, resulting in a deprived antioxidant system [80]. 

Majeed et al. (2021) analyzed the selenium status in COVID-19 patients and healthy individuals of South India. The patients showed significantly lower selenium levels of 69.2 ± 8.7 ng/mL compared to 79.1 ± 10.9 ng/mL in healthy individuals. The study further revealed lower than optimum selenium levels in an otherwise healthy population, indicating that this micronutrient level is not optimum in the population studied [81]. Similarly, decreased serum selenium levels were found as a risk factor for COVID-19 infection in a population in Iran [82]. Thus, selenium status might be one of the risk factors determining SARS-CoV-2 infection outcome, particularly in populations where Se intake is sub-optimal or low. Se status analysis in COVID patients provides important diagnostic information and suggests the need for adjuvant selenium supplementation in severely diseased and selenium-deficient patients.

### 6.2. Selenium on Severity and Mortality of COVID-19

A prospective observational study evaluated the micronutrient status, including selenium, in 84 patients diagnosed with COVID-19 categorized as mild, moderate, and severe based on symptoms. Patients with severe and critical symptoms have low oxygen saturation levels, septic shock, respiratory failure, and/or multiple organ dysfunction/failure. The serum Se status was 47.07 ± 20.82, 47.36 ± 25.6, and 29.86 ± 11.48 ng/mL in the mild, moderate, and severe disease groups, respectively, indicating the potential adverse role of selenium deficiency on COVID-19 [83]. A prospective observational study in Russia measured serum selenium status in 150 COVID-19 patients and 43 healthy participants. The serum selenium levels were significantly lower among severe and moderate cases compared to controls, but no significant difference was found between mild cases and controls. The study also indicated that reduced Se levels were inversely correlated with fever, lung damage, and inflammation and positively correlated with SpO2 in COVID-19 patients [84]. Similarly, a study conducted in 50 hospitalized COVID-19 patients in South Korea also showed that deficiency of selenium decreases the immunity against COVID-19 and causes progression to severity. Selenium deficiency was observed in 42% of patients with severe disease in South Korea [78]. In another cross-sectional study conducted in Belgium, the trace elements (Se, Zn, Fe and Cu, GPX3 activity and selenoprotein P (SELENOP) levels were determined in the serum of COVID-19 patients. It was observed that all the COVID-19 patients who succumbed to the infection had selenium deficiency with concentrations in the range of 23–64 ng/mL. Disease severity and length of hospital stay were found corelated to low selenium and SELENOP at the time of admission [85]. The abnormal concentrations of trace minerals, including selenium in urine, were associated with severe illness and fatal outcomes of COVID-19 [86].

A cross-sectional study was conducted in COVID patients in Germany in which total serum selenium, the activity of GPx-3 and SELENOP were assessed. The study revealed a significantly lower selenium status in the non-survivors compared to the survivors with respect to all three biomarkers of selenium. A time-resolved analysis of changes in selenium status of surviving vs. deceased COVID-19 patients showed that selenium status in survivors tended to recover and increase during hospital stay, whereas no such positive development was observed in the non-survivors [43]. The same authors also observed that the SELENOP levels along with serum Zn concentrations and age could be a reliable indicator of survival in COVID-19 [87]. Similarly, systemic oxidative stress status was found to be dangerously altered in critically ill COVID-19 patients who were admitted to the ICU. Increased lipid peroxidation and deficient antioxidant vitamins and minerals, including selenium, were observed in hospitalized COVID-19 patients with severe pneumonia [88]. Thus, these studies highlighted the exacerbating effects of selenium deficiency on COVID-19 severity, which can benefit from dietary selenium supplementation.

### 6.3. Selenium on the Severity of COVID-19 in Pregnant Women

Pregnancy is a state that makes women predisposed to viral infections due to the changes in their immune and cardiopulmonary systems. During the outbreak of H1NI in 2009, almost 1% of the patients infected with influenza A subtype H1N1 virus were pregnant women, resulting in high mortality [89]. Similarly, some strains of the coronavirus family also brought about severe complications during pregnancy, including admission to ICU and death [90,91]. The uncertainty of risk factors of COVID-19 in pregnancy impacted the pregnant individuals’ mental health negatively. Several studies worldwide have proposed that pregnant women are no more likely to contract COVID-19 than other healthy adults. However, there is a consistent association between pregnant individuals with COVID-19 and higher rates of adverse outcomes, including maternal mortality, preeclampsia, and premature birth, compared to those without COVID-19 [92]. 

Maternal selenium deficiency adversely affects not only the growth of the fetus but also the health and cognitive functions of infants during early stages of life. Selenium deficiency was associated with altered placental transport and is considered a risk factor for pregnancy-induced hypertension [93,94]. Generally, serum selenium levels decrease during pregnancy, while COVID-19 causes further reductions. Erol et al. (2021) showed significantly lower serum selenium levels of pregnant women with COVID-19 than the healthy group in the second and third trimesters [95]. Maternal selenium level was found to be correlated with blood cell count and C-reactive protein (CRP) levels in different stages of pregnancy, which indicates that serum selenium level deficiency affects immunity and CRP level. It was also observed that maternal selenium level also showed a correlation with D-dimer and IL-6 level in COVID-19 pregnant women, and hence, selenium status may be a preventative/predictive factor for COVID-19 severity, inflammation, and thrombosis [95]. 

## 7. Selenium Supplementation in COVID-19 Patients

**Figure 4:**
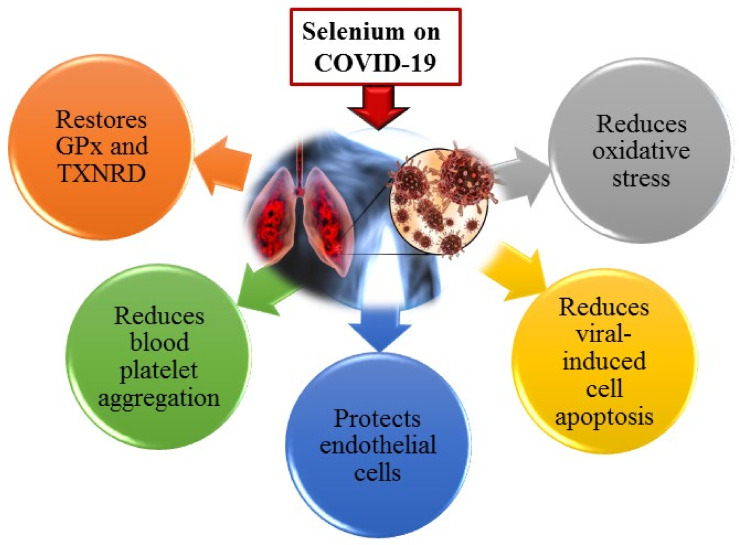
Effects of selenium supplementation on COVID-19. Adequate selenium levels reduce oxidative stress by restoring antioxidant enzymes, reduce cell death and coagulation pathways, and protect endothelial cells, thus having an overall protective effect on lungs and other organs.

Nutritional supplements, oriented to improve immune responses and defensive mechanisms against infections, have become a prime research focus during the pandemic. Many herbal and nutritional supplements have been analyzed to find out the potent ones that can reduce the progression of the infections where selenium is among the top priorities. The meta-analysis showed that parenteral selenium supplementation could reduce the overall mortality and duration of hospital stay in critically ill patients due to its antioxidant, anti-inflammatory, and antiviral properties (Figure 4) [96]. 

A single-center, randomized, double-blind, placebo-controlled study was conducted in 72 volunteers to analyze the effects of a synergistic combination of active ingredients containing selenium (100 µg/day) after vaccination against influenza or COVID-19. The mean levels of CD4+ T, CD3+ T, and CD8+ T lymphocytes showed a significant increase following the booster dose of the COVID-19 vaccine in the supplement group compared to the placebo. Serum selenium level was also increased in subjects treated with the active product without any observed adverse events [97]. Similarly, sodium selenite was reported to have antiviral activity due to its oxidizing capabilities. Therefore, this simple chemical compound can potentially be used in the recent battle against coronavirus epidemic [98].

Majeed et al. (2021) investigated the safety and efficacy of an active nutritional supplement, a combination of active herbal extracts and micronutrients named ImmuActive^TM^, in COVID-19 patients admitted to the hospital. A randomized, double-blind, placebo-controlled, multicenter, two-arm study assessed the effectiveness of the preparation containing selenium (40 µg/day) along with other ingredients as an adjunct therapy for COVID-19 patients for 28 days or until the discharge from hospital/COVID-19 care center or transfer to ICU, whichever was earlier in comparison to placebo. The subjects receiving selenium treatment had better therapeutic responses and reduced COVID-19 symptoms than placebo. Selenium supplementation lowered the mean duration for reducing COVID-19 severity by one unit, from 3.36 to 2.35 days compared to the placebo group Supplementation of ImmuActive^TM^ significantly reduced the duration of hospital stay and the number of days required to become coronavirus negative. Moreover, the supplementation was found safe without any adverse effects on health, indicating the formulation’s effectiveness to be used as an adjunct therapy for COVID-19 infection [99].

## 8. Conclusions

The management of COVID-19 is clinically challenging and controversial due to the lack of proven treatment. A better understanding of the immune and inflammatory responses during SAR-CoV-2 infections is essential to develop adequate therapeutic approaches. However, preclinical, and clinical studies have undoubtedly proven selenium as an important factor determining the severity of viral infections, especially respiratory viral diseases. Hence, selenium status can be considered an additional risk factor of SARS-CoV-2 infection, particularly in populations where selenium intake is sub-optimal or low. However, further clinical trials are required to design an effective treatment strategy for COVID-19 that includes selenium supplementation as an adjunct therapy.

## Data Availability

Not applicable.
